# Digital Health Interventions for Chronic Wound Management: A Systematic Review and Meta-Analysis

**DOI:** 10.2196/47904

**Published:** 2024-07-16

**Authors:** Xinrui Bai, Hongyan Zhang, Yanxia Jiao, Chenlu Yuan, Yuxia Ma, Lin Han

**Affiliations:** 1 Evidence-Based Nursing Centre School of Nursing Lanzhou University Lanzhou, Gansu China; 2 Department of Nursing Gansu Provincial Hospital Lanzhou, Gansu China

**Keywords:** chronic wounds, digital health interventions, wound healing, meta-analysis, systematic review, digital technologies, mobile health, eHealth, telemedicine, telehealth

## Abstract

**Background:**

Digital health interventions (DHIs) have shown promising results for the management of chronic wounds. However, its effectiveness compared to usual care and whether variability in the type of intervention affects wound outcomes are unclear.

**Objective:**

The main objective was to determine the effectiveness of DHIs on wound healing outcomes in adult patients with chronic wounds. The secondary objectives were to assess if there was any variation in wound healing outcomes across the various types of DHIs.

**Methods:**

In total, 9 databases were searched for the literature up to August 1, 2023. Randomized controlled trials (RCTs), cohort studies, and quasi-experimental studies comparing the efficacy of DHIs with controls in improving wound outcomes in adult patients with chronic wounds were included. Study selection, data extraction, and risk of bias assessment were conducted independently by 2 reviewers. We assessed the quality of each RCT, cohort study, and quasi-experimental study separately using the Cochrane risk of bias tool, ROBINS-I, and the Joanna Briggs Institute Critical Appraisal tools checklists. Relative risks (RRs) and 95% CIs were pooled using the random effects model, and heterogeneity was assessed by the *I^2^* statistic. Subgroup analysis and sensitivity analysis were also performed.

**Results:**

A total of 25 studies with 8125 patients were included in this systematic review, while only 20 studies with 6535 patients were included in the meta-analysis. Efficacy outcomes in RCTs showed no significant differences between the DHIs and control groups in terms of wound healing (RR 1.02, 95% *CI* 0.93-1.12; *P*=.67) and all-cause mortality around 1 year (RR 1.08, 95% *CI* 0.55-2.12; *P*=.83). Compared with the control group, the use of DHIs was associated with significant changes in adverse events (RR 0.44, 95% *CI* 0.22-0.89; *P*=.02). Subgroup analysis suggested a positive effect of the digital platforms in improving wound healing (RR 2.19, 95% CI 1.35-3.56; *P*=.002). Although meta-analysis was not possible in terms of wound size, cost analysis, patient satisfaction, and wound reporting rates, most studies still demonstrated that DHIs were not inferior to usual care in managing chronic wounds.

**Conclusions:**

The findings of our study demonstrate the viability of adopting DHIs to manage chronic wounds. However, more prominent, high-quality RCTs are needed to strengthen the evidence, and more detailed clinical efficacy research is required.

**Trial Registration:**

PROSPERO CRD42023392415; https://tinyurl.com/4ybz6bs9

## Introduction

Chronic wounds have a substantial impact on individual health, society, and health systems worldwide [1], with studies showing that the global prevalence of chronic wounds is estimated to be 1.67 per 1000 population [2]. A commonly used definition, labeling chronic wounds as wounds that “fail to proceed through an orderly and timely process to produce anatomic and functional integrity” [3]. Chronic wounds are classified by etiology and include, but are not limited to, lower extremity venous ulcers, neurological ulcers, diabetic foot ulcers, pressure injuries, and arterial ulcers [4], and these underlying pathologic factors often hinder or delay the healing process, resulting in significant negative impacts on the physical, emotional, and social well-being of patients. Many patients develop infections due to poor chronic wound management, experiencing increased pain, delayed wound healing, and even wound rupture and foul odors [5-7], imposing humanistic burdens (eg, health-related quality of life [8,9]) and economic costs (including direct expenditures, such as medical bills, and indirect lost productivity, such as sick leave and early retirement [10]) on both the patients themselves and society. It is crucial to highlight that there is no agreement on a specific healing period for chronic wounds, which means there is no set timeline for wound healing or when a wound becomes chronic; as a result, those suffering often require prolonged care [11].

Traditional wound care is mainly done in hospitals or specialized treatment facilities, and it is limited by time and location, treatment space, patients’ financial status, and the scope of the medical center’s services [10,12]. Patients, for example, frequently need to plan ahead of time for treatment, and those who reside in remote regions or have restricted mobility may face greater burdens [13]; so, many patients with chronic wounds remain without adequate wound management options. Consequently, even with the current advancements in in-hospital chronic wound management, it is still a vital challenge for the health care sector to address how to deliver a wound management program for patients with access challenges that is no less than the quality of in-hospital care without placing a greater financial burden on patients.

Digital health interventions (DHIs) have been recognized by the World Health Organization South-East Asia Regional Organization (WHO-SEARO) for their role in improving access to primary health care, may be a promising option for overcoming these barriers. According to the WHO definition, DHI is a discrete functionality of digital technology that is applied to achieve health objectives [14]. DHIs included, but were not limited to, devices used to deliver the intervention, such as mobile phones, mobile apps, portable tablets, web-based platforms, and activity trackers. DHIs are widely used for assessment, education, and symptom management in patients with a variety of disorders such as cancer, diabetes, stroke, and attention-deficit/hyperactivity disorder (ADHD) [15-19], with promising results in chronic wound management [20]. Remote consultation and follow-up via phone and email connect home-care nurses to wound experts, increasing the likelihood of wound healing [21-23]. The wound digital platform designed for inpatients has shown positive intervention outcomes [24,25]. Meanwhile, a novel study [26] developed the framework of the digital nursing quality management model and used digital wound care as an example in the conceptualization process, heralding the great potential of DHIs in the field of chronic wound management. Previous meta-analysis shows that DHIs show no inferiority in randomized trials compared with traditional face-to-face care. However, these meta-analyses have limitations: either only chronic wounds of 1 etiology have been considered [27,28], or they have looked at in control groups, such as community-based or nursing home–based interventions [29], whereas no studies have yet noted differences in DHIs.

Thus, an updated meta-analysis is warranted. The primary aim of this study is to assess the efficacy of DHIs for chronic wound management versus usual care. Our secondary aim is to explore whether and how modifiable types of DHIs (eg, digital platforms, telemedicine, or follow-up by telephone and email) affect chronic wound healing outcomes. These insights serve to inform existing or novel chronic wound-targeted treatment protocols and develop optimal treatment options for the benefit of patients.

## Methods

This systematic review is reported in accordance with the PRISMA (Preferred Reporting Items for Systematic Reviews and Meta-Analyses) 2020 statement (Multimedia Appendix 1) [30]. Before the start of the study, the review protocol was registered in PROSPERO (International Prospective Register of Systematic Reviews; CRD42023392415).

### Definitions and Categories of DHIs and Usual Care

The theoretical definition of DHI refers to a discrete functionality of digital technology that is applied to achieve health objectives. Telemedicine, digital platforms, and mobile phone and SMS text messaging follow-up are all examples of DHIs [31].

Although the definition of usual care has not been standardized, it can include the routine care received by patients for prevention or treatment of diseases [32]. In this study, in addition to regular hospital care, usual care forms consisted of the following 3 main categories and their collocated use: outpatient clinics, primary care, and home care. The operational definitions or meanings of several types of DHIs and usual care covered in this paper are explained in Table 1.

**Table 1:** Operational classification and definitions of digital health interventions and usual care.

Category			Operational definition or meaning
**Digital Health Intervention^a^**			
	Telemedicine	The use of electronic technology for information and communication by health care professionals with patients (or caregivers), with the objective of providing and supporting medical care to patients when they are away from health care institutions [33].	
	Digital platform	Intermediaries that enable 2 or more customer or supplier and user groups to interact [34].	
	Follow-up by telephone and email	A method in which health care professionals evaluates a patient’s health status along with offering periodic care using verbal descriptions over the phone or electronic information sent via mail.	
**Usual care**			
	Outpatient clinic	One of the most important departments of the hospital, where most elective care trajectories begin, with a consultation between a care provider and a patient [35].	
	Primary care	The provision of integrated, accessible health care services by clinicians who are accountable for addressing a large majority of personal health care needs, developing a sustained partnership with patients, and practicing in the context of family and community [36].	
	Home care	Medical and paramedical services delivered to patients at home [37].	

^a^Only the digital health intervention categories addressed in this systematic review were explained.

### Search Strategies

The 2 researchers XB and HZ performed an independent electronic search in PubMed, Web of Science, EMBASE, Cochrane Library, CINAHL, China Knowledge Resource Integrated Database, Wanfang Database, Weipu Database, and Chinese Biomedical from their inception to August 1, 2023. The search strategy was developed using the PICO search framework (ie, patient, intervention, comparison, outcome, and study design) and the search terms were divided into 3 categories: patients with chronic wounds (population), digital health (intervention), and wound status (outcome). Each category was combined with MeSH (Medical Subject Headings) terms and natural language (Multimedia Appendix 2).

### Eligibility Criteria

The inclusion criteria of this study, which followed the PICOS (Population, Intervention, Comparison, Outcomes, and Study) design framework [38], were as follows: (1) Population: adults aged 18 years or older with chronic wounds of any type and severity. Chronic wound was defined as a wound that “fail to proceed through an orderly and timely process to produce anatomical and functional integrity” and follow the 4 major classifications of the Wound Healing Society, ie, pressure ulcers, diabetic foot ulcers, venous ulcers, and arterial insufficiency ulcers [6]; (2) Interventions: study interventions must have used a DHI to capture, assess, create, or communicate wound status in patients with chronic wounds. DHIs were defined as “health promotion approaches aided by various digital technologies,” such as remote consultations, mobile applications, web-based platforms, mobile phones, wearables, SMS, and email; (3) Comparison: studies that assigned participants into either an experimental group or a control group including usual, routine, and conventional care, or waitlist as defined by the original research; (4) Outcomes: the primary outcome was the state of chronic wound healing (eg, wound healing, healing time, and change in wound size), and secondary outcomes include all-cause mortality, adverse events, patient satisfaction, and certain wound-specific scoring metrics; and (5) Study design: randomized controlled trials (RCTs), cohort studies, and quasi-experimental studies published in English and Chinese.

Articles were excluded if their main objective was to assess the acceptability of a newly developed DHI among patients or if the study did not contain a control group. Conference proceedings, magazines, news, electronic resources and reports, theses, dissertations, abstracts, editorials, and systematic reviews were also excluded.

### Study Selection

**Figure 1:**
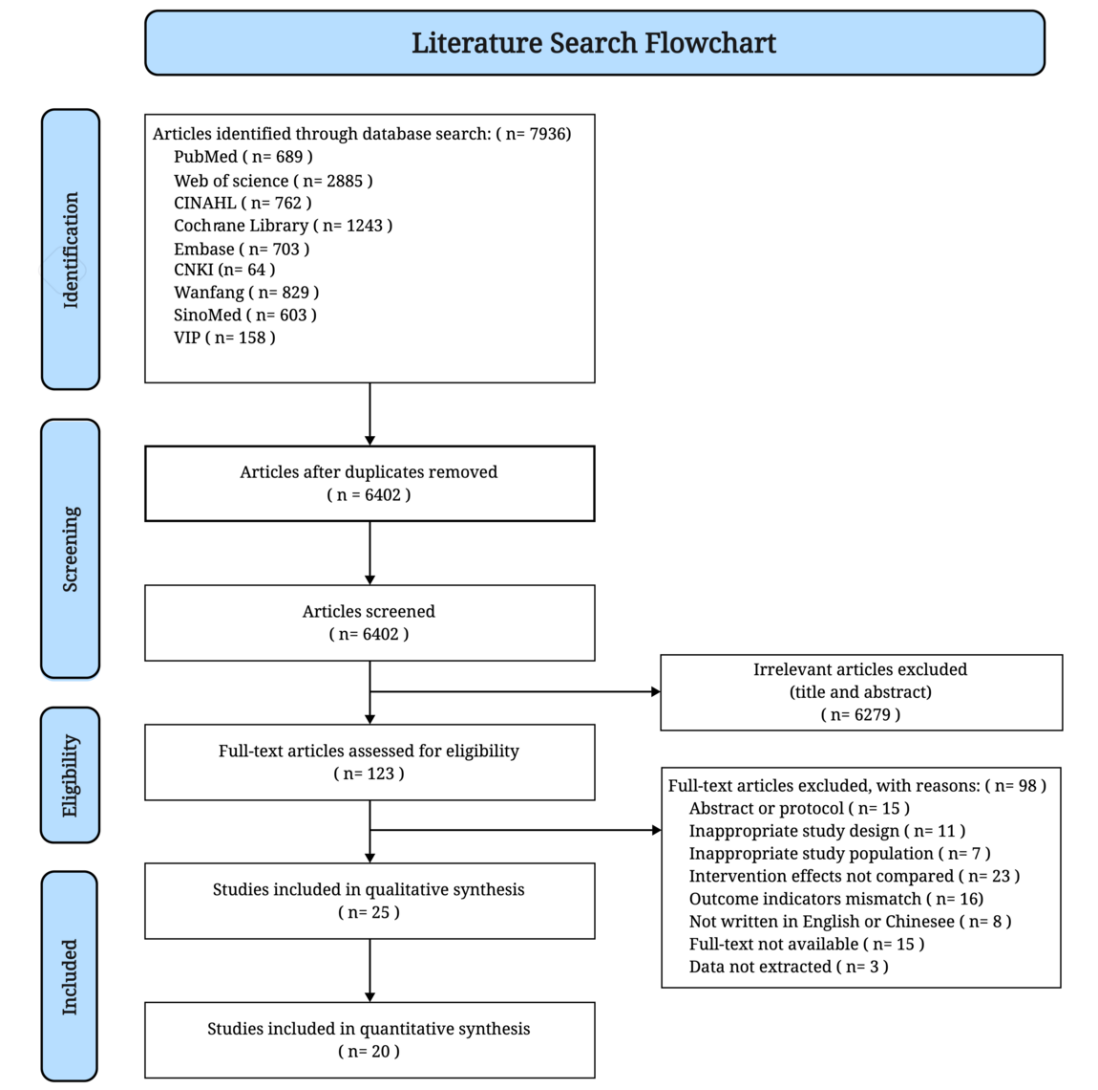
PRISMA (Preferred Reporting Items for Systematic Reviews and Meta-Analyses) flow diagram. CNKI: China Knowledge Resource Integrated Database; VIP: Weipu Database.

Initially, search duplicates were removed using the reference manager tool EndNote (version X9.3.3; Clarivate). For final inclusion, each study was assessed independently by 2 researchers (XB and HZ), first by screening the title and abstract, and then through a full-text review. Disagreements on the selection of records between the 2 researchers were resolved by team discussion or by a third researcher (LH). In addition, we manually searched the reference lists of the included studies for additional studies. The PRISMA flow diagram was used to illustrate the study selection (refer to Figure 1).

### Data Extraction

XB and HZ independently extracted the data using a pre-designed form created with Excel. Disagreements were resolved through discussion or with assistance from a third reviewer (LH), if necessary. From each study, we extracted information about study details (eg, title, author, year, and country), study design (type of study, aims, sample methods, and inclusion or exclusion criteria), participants’ characteristics (number of persons surveyed, population characteristics, ie, age, gender, and demographics), specific wound data and complications (diagnostic methods, ulcer specifications including stage), and outcomes (eg, wound healing, healing time, wound size change, adverse event, all-cause mortality, cost, and patients’ satisfaction).

### Assessment of Risk of Bias

The risk of bias for each study was assessed by 2 independent reviewers (XB and HZ). For RCTs, the Cochrane risk-of-bias tool [39] was used to assign a “high risk,” “unclear risk,” or “low risk” according to 5 domains: (1) sequence generation, (2) allocation concealment, (3) blinding, (4) incomplete outcome data, and (5) selective outcome reporting. We used ROBINS-I [40] for cohort studies, which assesses 7 types of bias from confounding variables, selection of participants, measurement classification of interventions, deviations from intended interventions, incomplete outcome data, outcome assessment, and selective outcome reporting. For quasi-experimental studies, the checklists of the Joanna Briggs Institute Critical Appraisal tools were used, comprising 9 items that can be rated yes, no, unclear, or not applicable.

### Statistical Analysis

When possible, outcome data were analyzed quantitatively by calculating a pooled effect of different studies. A meta-analysis was performed when ≥2 studies with available data investigated the same outcome; otherwise, the outcomes were presented narratively. Considering the expected clinical heterogeneity among the included studies, the random-effects model was used to estimate the overall effect size and 95% *CI*. The pooled analysis was presented as a risk ratio (RR). The effect of the intervention on continuous outcomes was expressed as standardized mean difference (SMD) for outcomes reported with different measures and with 95% *CI* [41].

Heterogeneity among studies was estimated Cochran *Q* test and the *I*^2^ statistic (*I*^2^>50% indicated substantial heterogeneity) [42]. We also performed sensitivity analyses to examine the robustness of the results. Sensitivity analysis was carried out by removing a single study at a time to see how it impacted the overall estimate. For chronic wounds’ outcomes, the forest plots were also constructed. When the number of included studies was more than 10 [43], the graphical symmetry of the funnel plot and the Egger test statistic were used to detect possible publication bias [44], because the power of the test is lower when the number of studies is small. In the absence of publication bias, the funnel plot is expected to be symmetrical, and the *P* values of Egger’s test are >.05 [45,46].

In addition, RCT and observational data were analyzed separately, and exploratory subgroup analyses were carried out for different modalities of DHI. All analyses were performed with RevMan (version 5.4.1; The Cochrane Collaboration) and Stata (version 15.0; StataCorp; XB and HZ).

## Results

Out of a total of 7936 potential articles identified in the literature search, 123 studies were selected for a full review. Finally, the systematic review included 25 studies involving a total of 8125 patients: 14 (56%) presented the results of RCTs [22,23,47-58], 6 (24%) cohort studies [21,59-63], and 5 (20%) quasi-experimental studies [24,25,64-66]. Figure 1 depicts the results of the study selection.

### Study Characteristics

In total, 25 studies were published between 2004 and 2023, with 9 (36%) conducted in China [24,25,50,54-56,64-66], 3 (12%) in Norway and France [52,53,57,58,60,61], and 2 (8%) in the United States [48,59], Australia [47,51], and Denmark [21,49], respectively. In addition, the following countries had 1 (4%) of the studies each, the United Kingdom [22], Canada [23], Sweden [62], and Israel [63]. Sample sizes varied across studies, ranging from the smallest sample of 26 subjects in Vowden and Vowden [22] to the largest of 1988 subjects in Wickstrom et al [62]. A total of 40% (10/25) of the studies were conducted on patients with chronic wounds of various etiologies [22,47,48,53,55, 56,60-62,66], 32% (8/25) focused on patients with pressure injuries [23-25,51,54,57,64,65], 16% (4/25) targeted patients with diabetic foot ulcers [49,52,58,59], 4% (1/25) recruited patients with lower extremity ulcers of various etiologies [63], 4% (1/25) included only patients with lower extremity venous ulcers [50], and a further 4% (1/25) excluded patients with pressure ulcers, surgical wounds, and cancer wounds [21].

Wound care in experimental groups varied, because of the clinical heterogeneity of DHIs between studies, with 15 (60%) studies collecting patients’ wound data via telemedicine, such as web-based programs and video conference [21,22,47-50, 53,54,57-59,61-63], 7 (28%) studies using the digital platform [24,25,55,56,64-66], and 3 (12%) studies using email and phone to facilitate the implementation. The follow-up periods were inconsistent among all studies and, where present, ranged from 3 to 35 months. In addition to hospitalization, the control group received wound usual care in a variety of settings, including outpatient clinics (14/25, 56%) [23-25,47-52,63-67], the home (3/25, 12%) [21,22,60], the community (2/25, 8%) [61,62], and home care combined with outpatient follow-up (1/25, 4%) [59]. With the exception of 2 (8%) studies, the remaining 23 (92%) studies were divided into 2 groups, where 3 groups were compared by Terry et al [48], group A (weekly visits with TM and wound care specialist consults), group B (weekly visits with weekly consults with WCS), and group C (routine care). Téot et al [53] divided the control group into group 1 (telemedicine), group 2a (home care), and group 2b (clinic care). Details of all 25 studies are summarized in Table S1 in Multimedia Appendix 3 [21-25,47-66].

### Risk of Bias

Details of the assessment of the risk of bias are presented in Tables S2-S4 in Multimedia Appendix 4 [21-25,47-66]. Of the 14 RCTs, 4 (29%) RCTs were assessed as high risk in the “other bias” option, owing to unequal baseline characteristics [22,48,52,53], and 6 (43%) studies [49-51,55,57,58] demonstrated a low risk of bias, with bias in ≤1 domain.

The assessment result for cohort studies revealed that 2 (33%) studies [21,60] had a moderate risk of bias, while 4 (67%) studies [59,61-63] had a high risk of bias.

Finally, for the quasi-experimental study, all study items related to the integrity of follow-up were rated as not applicable, and the items were rated as unclear mostly because it was unknown whether the other measures received by the groups were identical.

### Outcome Analysis

#### Wound Healing

**Figure 2:**
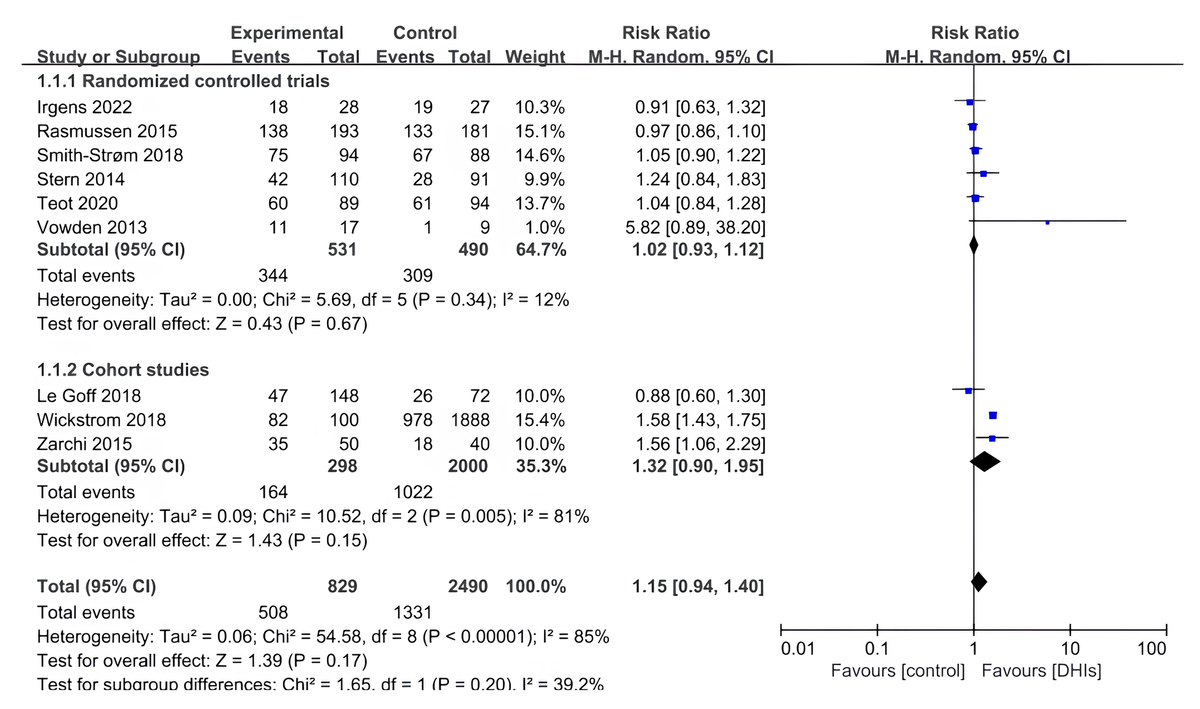
Forest plot of digital health interventions (DHIs) on wound healing around one year (different study types).

Of the 25 studies, 10 (40%) studies [21-23,48,49,52,53,57,61,62] reported wound healing around 1 year, including 7 (28%) RCTs and 3 (12%) cohort studies. One (4%) RCT [48] was not included in the quantitative synthesis because of the uneven distribution of severity of wounds among groups. Pooled data in 9 (36%) studies revealed no significant difference in wound healing (RR 1.15, 95% CI 0.94-1.40; *P*=.17; *I*^2^=85%; Figure 2), and the finding for the pooled is consistent in RCTs (RR 1.02, 95% CI 0.93-1.12; *P*=.67; *I*^2^=12%) and cohort studies (RR 1.32, 95% CI 0.90-1.95; *P*=.15; *I*^2^=81%).

A total of 12% (3/25) of the studies reported wound healing at 3 months, including 1 RCT and 2 cohort studies [50,59,60]. Pooling the data showed that wound healing at 3 months seems superior in the DHIs group than the control group with usual care, but no statistically significant difference was observed (RR 1.44, 95% CI 0.51-4.05; *P*=.49; *I*^2^=75%; Multimedia Appendix 5).

Additionally, one study [58] found that the intervention group’s ulcer healing rate was 62.1%, which was greater than the control group’s rate of 52.4%. Furthermore, the study by Wu and Fu [65] showed an increase in wound healing rate after the intervention; Santamaria et al [47] reported positive wound healing rates per week in the intervention group as well as negative rates per week in the control group; another study [63] defined a positive outcome as at least 50% ulcer closure. None of these 4 (16%) studies were appropriate for inclusion in the quantitative analysis.

**Figure 3:**
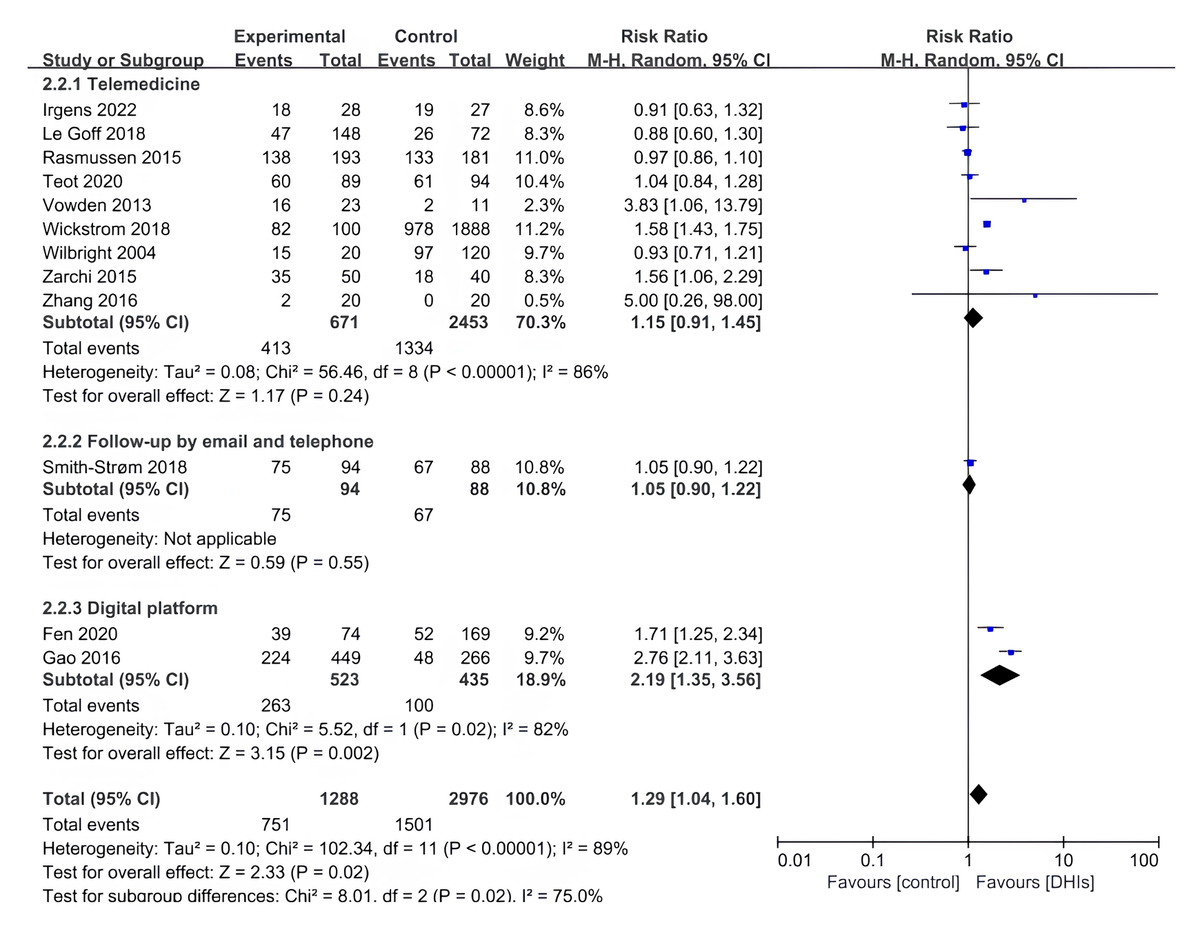
Forest plot of digital health interventions (DHIs) on wound healing around 1 year (subgroup of intervention types).

The studies were separated into 3 subgroups: telemedicine, email and telephone follow-up, and digital platform, based on the clinical heterogeneity of the types of DHIs in the intervention groups. Providers also varied in the telemedicine model, with 73% (11/15) of the studies involved nurses as interveners [22,47,49,50,54,58-63], 20% of studies involved wound care specialists (3/15) [21,48,53] and a further 7% (1/15) with a multidisciplinary team responsible for the intervention [57]. Subgroup data demonstrated a statistically significant difference between the DHIs and control groups when the digital platform was used (RR 2.19, 95% CI 1.35-3.56; *P*=.002; *I*^2^=82%; Figure 3). However, there was no significant change in wound healing in the groups with telemedicine (RR 1.15, 95% CI 0.91-1.45; *P*=.24; *I*^2^=86%).

#### Wound Healing Time

Of the 25 studies, 8 (32%) studies reported different forms of wound healing time [52,53,55,57-59,61,62], 12% (3/25) of the studies, including 2 RCTs and 1 cohort study, reported the mean and SD of wound healing time frames [52,53,59], and a further 12% (3/25) of the studies provided median wound healing times [57,61,62], with 2 studies demonstrating considerable effectiveness in the DHIs group [61,62], and 1 study demonstrating a shorter wound healing time in the control group compared to the DHIs group (*P*=.56). In a study (1/25, 4%) that tracked the effect of the intervention according to the size of the patient’s wounds, the results showed that the healing time of large, medium, and small wounds was shorter in the intervention group than in the control group (*P*<.05) [55]. It is also noteworthy that Dardari et al [58] found that wounds in the intervention group first showed improvement on day 21, much earlier than day 77 in the control group.

#### Wound Size

Of the 25 studies, 8% (2/25) of the studies consisting of in total of 168 patients reported a reduction of the wound area [51,60]. One of the studies [51] also noted that the DHIs group showed more considerable changes in wound depth. Another study [57] described the ulcer volume, indicating a mean reduction in ulcer volume in remote consultation was 79% versus 85% in usual care (*P*=.32).

#### Wound Reporting Rate

In total, 8% (2/25) of the studies reported wound reporting rate, all of which showed a significant increase following the use of a digital platform to monitor pressure injuries [24,65]. In addition, 1 pilot study (4%) noted in its results that the accuracy of screening and reporting of patients with pressure injuries from 97.26% to 100% after using the digital platform [64].

#### All-Cause Mortality

**Figure 4:**
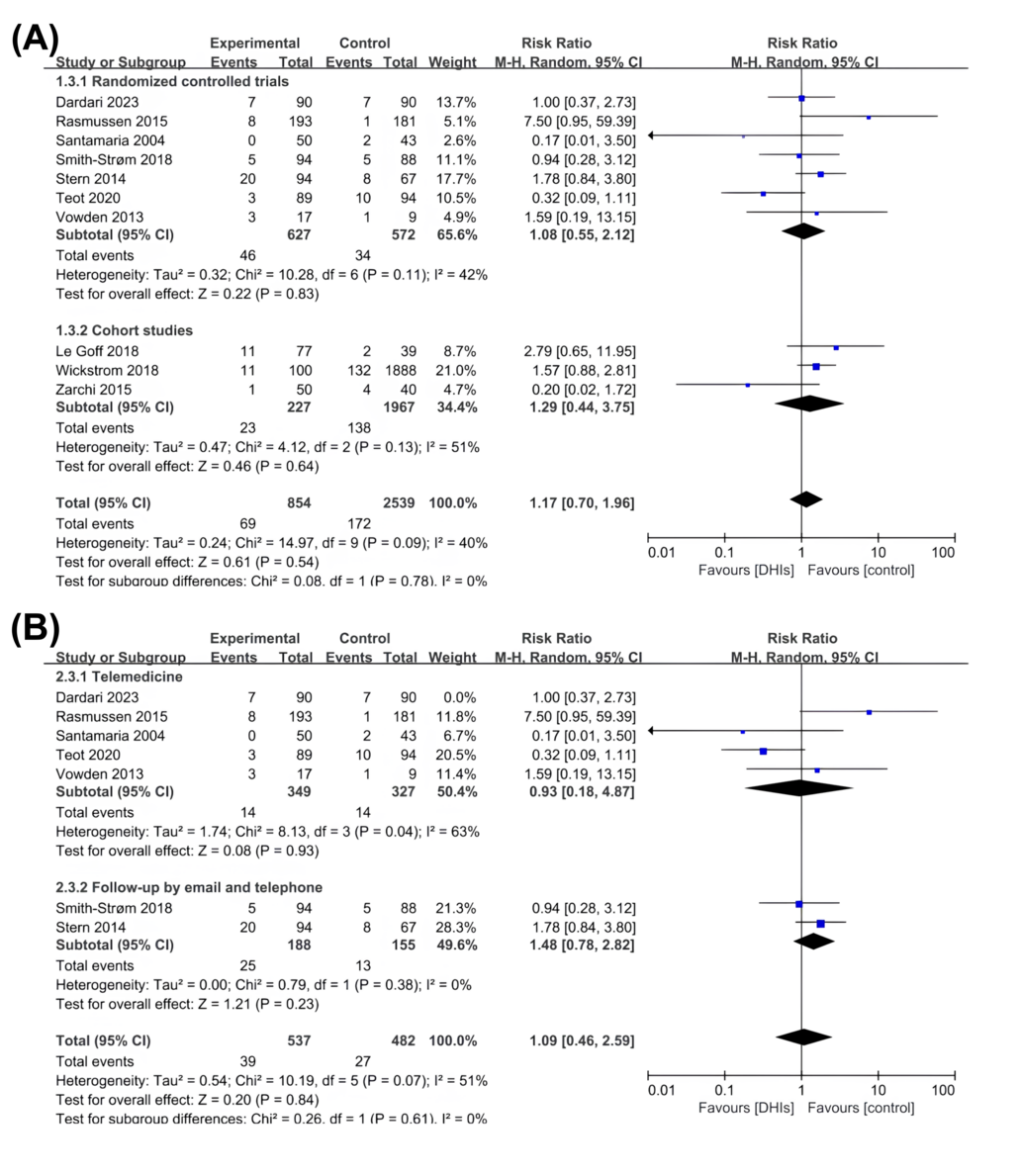
(A) Forest plot of digital health interventions (DHIs) on all-cause mortality. (B) Forest plot of DHIs on all-cause mortality (subgroup of intervention types).

Of the 25 studies, 40% (10/25) of the studies reported all-cause mortality around 1 year [21-23,47,49,52,53,58,61,62]. There was no significant difference in all-cause mortality between the DHIs and control groups (RR 1.17,95% CI 0.70-1.96; *P*=.54; *I^2^*=40%; Figure 4A). This finding is consistent in RCTs (RR 1.08, 95% CI 0.55-2.12; *P*=.83; *I*^2^=42%) and cohort studies (RR 1.29, 95% CI 0.44-3.75; *P*=.64; *I*^2^=51%). Subgroup analysis of RCTs reveals there was no statistically significant decreased risk of all-cause mortality in patients receiving telemedicine (RR 0.93, 95% CI 0.18-4.87; *P*=.93; *I*^2^=63%) and follow-up by email and telephone (RR 1.48, 95% CI 0.78-2.82; *P*=.23; *I*^2^=0%; Figure 4B).

#### Adverse Event

A total of 6 (24%) studies [47,49,52,56,58,62], including 5 (20%) RCTs and 1 (4%) cohort study, reported the incidence of adverse events (infections and amputations). A meta-analysis of all RCTs found no statistically significant difference in the incidence of adverse events between the DHIs and the comparator group in patients with chronic wounds (RR 0.56, 95% CI 0.29-1.11; *P*=.10; *I*^2^=77%) (Multimedia Appendix 6). Since 1 (4%) RCT clearly stated that none of the adverse events were attributed to the intervention [58], we attempted to remove this research from the meta-analysis and found that in the remaining 4 (16%) RCTs, the DHIs group had even fewer adverse events (RR 0.44, 95% CI 0.22-0.89; *P*=.02; *I*^2^=41%) (Multimedia Appendix 7). Another cohort study (1/25, 4%) revealed that the DHIs group had a 2% amputation rate compared to 1% in the control group [62].

#### Cost Analysis

A cost analysis of DHIs against controls was addressed in 8 (32%) studies [23,48,49,51,53,58,60,61], with 5 (20%) studies revealed a reduction in total costs [23,49,58,60,61]. One study attributed the significant reduction in costs in the intervention group to a shorter hospital stay due to telemedicine [58], and another study investigated transport costs and found that the DHIs group spent an average of approximately US $650 less per person on transport for wound care compared with usual care [53]. However, 2 (8%) studies also concluded that the total costs were higher in the DHIs group [48,51], but for different reasons; the study by Terry et al [48] pointed out that the increased cost was due to larger and more severe wounds in the telemedicine group of patients, while Arora et al [51] attributed the increased cost to the use of new technology and assistive devices in the intervention group.

#### Patient Satisfaction

Patient satisfaction was reported in only a few (3/25, 12%) studies [52,56,66], of which 2 (8%) studies used a self-administered satisfaction questionnaire and found a substantial improvement in patient satisfaction after using DHIs [56,66], and the remaining study (1/25, 4%), measured using the GS-PEQ (The Generic Short Patient Experiences Questionnaire) scale [52], found no significant difference between the DHIs and the control group in patient satisfaction (MD 0.07, 95% CI 0.10-0.24). Due to heterogeneous outcome measures, a quantitative evaluation was not possible.

#### PUSH-Score

In addition to the typical result indicators for chronic wound evaluation outlined above, we observed significant changes in the PUSH-score (Pressure Ulcer Scale for Healing score), which is used to assess pressure injury only, in the DHIs group in 3 (12%) studies [51,54,66], including 2 (8%) RCTs and 1 (4%) quasi-experimental study. Although the variability of the research designs prohibited us from doing a meta-analysis, all studies revealed that using a digital platform improved the PUSH-score in patients with pressure injuries.

### Publication Bias and Sensitivity Analysis

**Figure 5:**
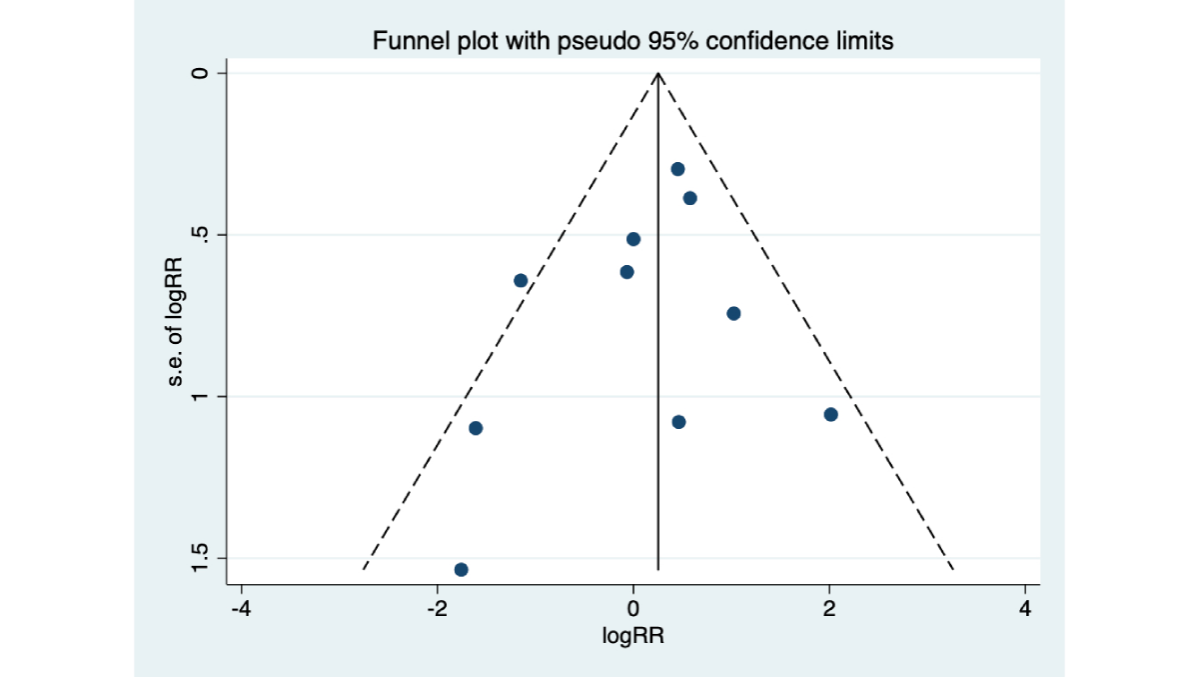
Funnel figure of test for publication bias. RR: relative risk.

The funnel plot for all-cause mortality underlying the meta-analyses was symmetrical, which reflected a low risk of publication bias (Figure 5), the Egger test also showed consistent result (*P*=.37).

**Figure 6:**
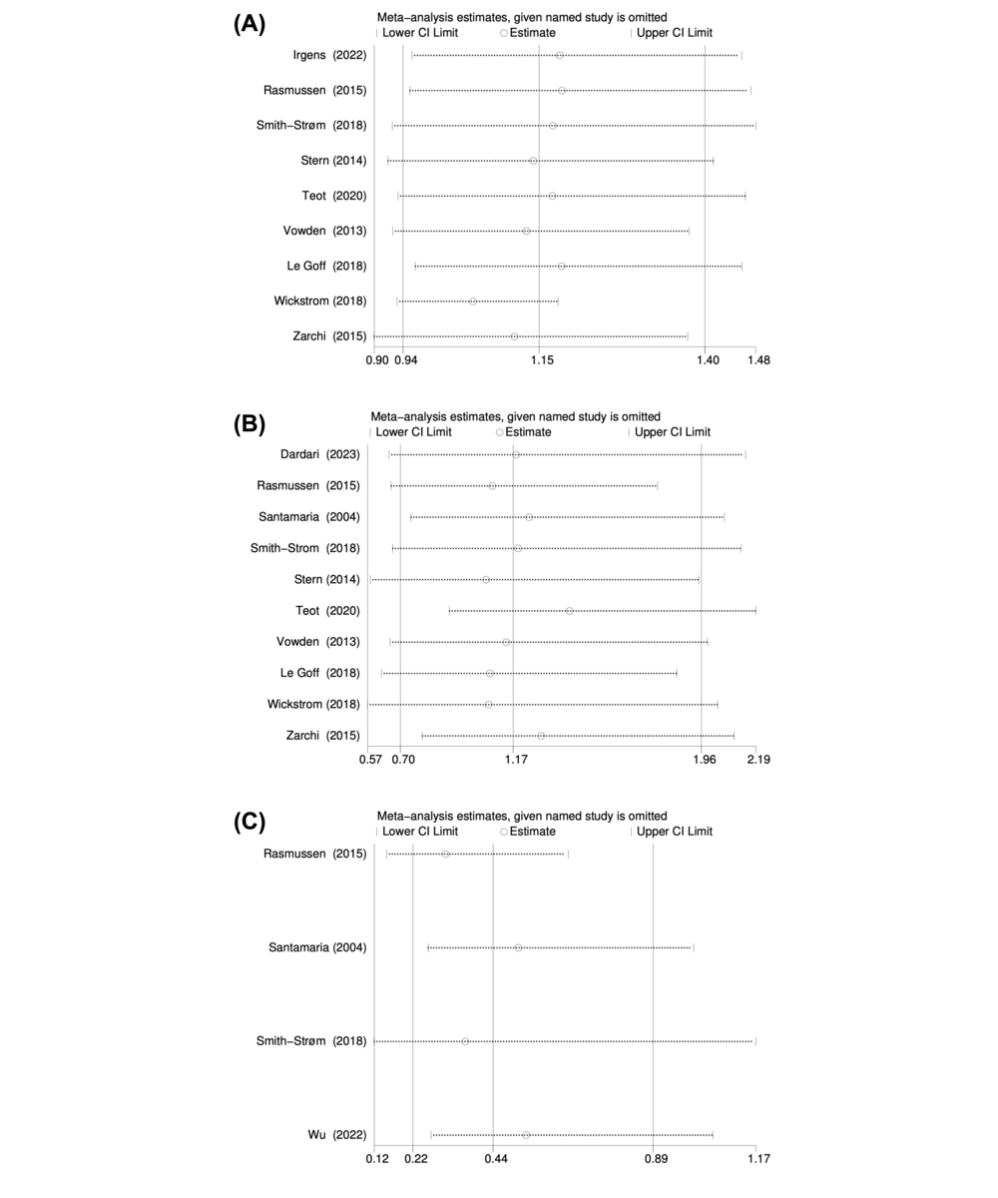
(A) Sensitivity analysis of wound healing. (B) Sensitivity analysis of all-cause mortality. (C) Sensitivity analysis of adverse event.

Sensitivity analysis was performed by omitting studies sequentially (Table 2). For wound healing around 1 year (Figure 6A), the pooled RR ranged from 1.05 (95% *CI* 0.93-1.18) to 1.19 (95% *CI* 0.95-1.47). For all-cause mortality (Figure 6B), the pooled RR ranged from 1.06 (95% *CI* 0.58-1.95) to 1.41 (95% *CI* 0.91-2.19). For adverse event (Figure 6C), the pooled RR ranged from 0.31 (95% *CI* 0.15-0.65) to 0.54 (95% *CI* 0.27-1.05). The results revealed that each outcome was relatively stable and would not change due to the elimination of a study; thus, the result of meta-analysis was robust.

**Table 2:** Sensitivity analysis of included studies.

Study omitted	RR^a^ (95% CI) for wound healing	RR (95% CI) for all-cause mortality	RR (95% CI) for adverse event
Irgens (2022)	1.18 (0.96-1.46)	—^b^	—
Rasmussen (2015)	1.19 (0.95-1.47)	1.09 (0.66-1.78)	0.31 (0.15-0.65)
Smith-Strøm (2018)	1.17 (0.93-1.48)	1.19 (0.67-2.12)	0.37 (0.12-1.17)
Stern (2014)	1.14 (0.92-1.42)	1.06 (0.58-1.95)	—
Teot (2020)	1.17 (0.94-1.46)	1.41 (0.91-2.19)	—
Vowden (2013)	1.13 (0.93-1.38)	1.14 (0.66-1.98)	—
Le Goff (2018)	1.19 (0.96-1.46)	1.08 (0.62-1.86)	—
Wickstrom (2018)	1.05 (0.93-1.18)	1.07 (0.57-2.03)	—
Zarchi (2015)	1.11 (0.90-1.38)	1.29 (0.79-2.09)	—
Dardari (2023)	—	1.19 (0.66-2.14)	—
Santamaria (2004)	—	1.24 (0.75-2.05)	0.51 (0.26-1.00)
Wu (2022)	—	—	0.54 (0.27-1.05)
Combined	1.15 (0.94-1.40)	1.17 (0.70-1.96)	0.44 (0.22-0.89)

^a^RR: relative risk.

^b^Not applicable.

## Discussion

### Principal Results

This review provides a synthesis of high-quality evidence pertaining to the efficacy of DHIs for chronic wounds with different etiologies. Altogether, 12 RCTs, 5 cohort studies, and 3 quasi-experimental studies were identified and included in this meta-analysis. The magnitude of the intervention effect varied across studies, influenced by factors such as the nature of the intervention, assessment methods, and intervention duration.

The meta-analysis showed that DHIs did not significantly differ from usual care in terms of wound healing and all-cause mortality over around 1 year, which is consistent with findings of a previous meta-analysis targeting the effectiveness of telemedicine for chronic wound management [29]. Nevertheless, we believe it may be too early to conclude that DHIs are as equally effective as usual care in improving wound healing due to the lack of a sufficient number of high-quality randomized controlled studies and the fact that digital technology is always evolving.

Our study also suggests that DHIs can reduce adverse events in chronic wound management, which is inconsistent with existing evidence. A previous meta-analysis showed no significant difference in the number of adverse events between the telemedicine and usual care groups [67]. This may be due to the inclusion of other forms of DHI in addition to telemedicine in our study, and it also lends support to the idea of exploratory subgroup analyses of intervention types.

Subgroup analyses demonstrated a beneficial effect of digital platforms for hospitalized patients on wound healing, implying differences in chronic wound management outcomes among intervention types, which may be linked to the characteristics and limitations of the technologies. Specifically, teleconsultation scenarios in the form of videoconferencing are excellent [68], but require high levels of equipment and patient finances [51,69]. Although telephone and email follow-ups are more concise and convenient [70], visual diagnosis of wounds is lacking and the effectiveness of the interaction is difficult to ensure; for example, nurses are frequently unable to validate that patients have read and understood the content of the email [71]. Furthermore, digital platforms used for in-hospital patient pressure injury evaluation and management have shown promise in improving patients’ PUSH-score, but evidence from these studies is limited. Through further analysis of studies reporting this outcome metric, we discovered that the significant improvement in patients’ PUSH-score is closely linked to the real-time tracking function of the digital platform. This feature effectively aligns with the PUSH scale’s dynamic assessment of pressure injuries, enabling the quantification of dynamic changes in patients’ wounds [72]. Additionally, the platform's built-in automated analytics function provides critical feedback, offering a reliable predictive basis for nurses to identify the risk of pressure injury development in patients [64,73]. However, whether digital platforms provide better intervention outcomes than other types of DHIs still need to be validated and explored in higher-quality studies.

Of note, few studies have considered the impact of patient age on the effectiveness of technological interventions, and the groups included in the studies were predominantly middle-aged and older people, but the fact is that digital health care is more accepted in younger age groups [74]. Cost is another component that must be considered. Several studies have combined different forms of digital health technology to positively impacted wound outcomes; yet, it remains to be seen whether the increased cost of care as a result of the combination of technologies reduces patient adherence to treatment, which in turn affects wound outcomes [74]. Similarly, unequal access, use, and knowledge of information and communication technology among patients may also affect the effectiveness of DHIs.

In addition to the issues mentioned above, a point of concern, however, was a lack of blinding in most studies. The nature of interventions made them difficult to blind participants or providers, as the conduct of interactions frequently necessitates that both parties understand what they are doing. Patients, for example, are required to prepare their own computers or visit certain specific websites to validate and register their personal identities when conducting real-time videoconferencing-supported wound diagnosis and follow-up, and it is difficult for doctors or nurses on the other end of the video to be unaware of the content and form of the intervention, which is closely related to their work experience and professionalism; when using mobile phones or SMS text messaging for interventions, patients and researchers frequently agree on the frequency of the intervention ahead of time and exchange contact information in case they miss or ignore the diagnostic content, care recommendations, or follow-up feedback. Future RCTs should address this aspect to strengthen the evidence on DHIs for the management of patients with chronic wounds.

### Strengths and Limitations

To our knowledge, this is one of the most comprehensive and up‐to‐date reviews and meta‐analyses to evaluate the effects of DHIs on chronic wound outcomes across a broad spectrum of the population, combining data from RCTs, cohort studies, and quasi-experimental studies. While there have been studies looking at the use of digital health technology in chronic wound management, this study is unique in that it notes the diversity of interventions and attempts to quantify the effects that different types of DHIs show in terms of improving wound outcomes. For example, an explorable link was discovered between digital platforms and changes in PUSH-score in patients with pressure injuries. This provides new ideas for future research on whether different types of digital intervention techniques can be coupled to various etiologic wound evaluation tools to improve intervention outcomes. Furthermore, studies included in this review cover a wide range of DHIs, which differ in terms of the persons engaged, the intervention management, and the technology used, and are applied to populations with various wound etiologies, allowing the results to be generalizable.

Despite the clinical implications and strength of our review, certain limitations need acknowledgment. First, few researchers have specified the wound staging of included patients, which prevents us from drawing firm conclusions about this. Additionally, differences in participants, intervention contents, methods, frequency, time, and measurements in the control group also resulted in heterogeneity. Moreover, due to the significant variation in intervention design, it was difficult to extract and classify interventions in a very standardized way. Even though we divided it into 3 types of intervention subgroups, the interventions are still not standardized within each subgroup, and many parameters are implicitly variable, which may influence the results of our review. Finally, it must be explained that we included other study types in the meta-analysis in addition to RCTs, which is not recommended by the official guidelines for meta-analysis. Therefore, to overcome this limitation, we have clearly categorized and described in detail the results of the RCTs and non-RCTs included in this paper to improve the clarity and reference value of the results.

### Implication for Practice and Research

The results of the subgroup analyses point to the benefits of digital platforms for chronic wound management in hospitalized patients, but a limitation that cannot be ignored is that most of the studies were quasi-experimental and the platforms they used varied in terms of both structural design and technological quality. Therefore, future studies should be based on evidence-based practice, attempt to develop a digital platform that can be replicated on a large scale, and conduct more RCTs, considering the context and needs of the population, such as the acceptability of the technology, economic disparities, and the use of other services. At the same time, given the association between age and digital health literacy, it is necessary to provide interventions for each age group to clearly confirm effects in future studies.

In addition to these clinical implications, there are several possible directions for further research. Considering the heterogeneity of the interventions and the wound etiology, we recommend further research to investigate whether there are certain associations between the types of digital health interventions and patient characteristics to provide a valid reference for the clinical construction of a systematic digital wound management program, such as differences in the effectiveness of the same interventions for patients with wounds of different etiologies, and the relationship between patients’ digital health literacy and the effectiveness of the interventions. Moreover, regarding the review process we mentioned in the principal results section, we found that only a few studies blinded wound therapists, nurses, or patients. We recommend that future studies consider using existing high-quality patient digital information collection programs or web-based data platforms and rationally using the automated analytical capabilities of the technology to conduct single-blind experiments in which patients with comparable baseline information are randomly grouped with the consent of the patient, and therapists are implemented blinding of assessors by performing software-based outcome measures for all primary and secondary outcomes and automatically storing patient self-reported data. Data review should be done in a blinded manner until analyses were performed, and data analysis was also done using blinded subgroups to improve the quality of evidence generated by the study.

### Conclusions

In summary, this study suggests that DHIs are effective for chronic wound management, but the jury is still out on who is better between them and usual care. We also found indications that digital platforms can help with chronic wound management in hospitalized patients, warranting further investigations. Moreover, future high-quality research is needed, to identify factors contributing to improved patient-centered interventions with better care outcomes, as well as more careful consideration of individual patient characteristics.

## Appendix

Multimedia Appendix 1 PRISMA (Preferred Reporting Items for Systematic Reviews and Meta-Analyses) checklist.

Multimedia Appendix 2 Search strategy for all databases.

Multimedia Appendix 3 Characteristics of the included studies.

Multimedia Appendix 4 Risk of Bias.

Multimedia Appendix 5 Forest plot of digital health interventions on wound healing around three months (different study types).

Multimedia Appendix 6 Forest plot of digital health interventions on adverse event (randomized controlled trials).

Multimedia Appendix 7 Forest plot of digital health interventions on adverse event (adjusted version for randomized controlled trials).

## Abbreviations

ADHD: attention-deficit/hyperactivity disorder
DHI: digital health intervention
MeSH: Medical Subject Headings
PICOS: Population, Intervention, Comparison, Outcomes, and Study
PRISMA: Preferred Reporting Items for Systematic Reviews and Meta-Analyses
PROSPERO: International Prospective Register of Systematic Reviews
RCT: randomized control trial
RR: relative risks
SMD: standardized mean difference
WHO-SEARO: World Health Organization South-East Asia Regional Organization

## Data Availability

The authors confirm that the data supporting the findings of this study are available within the article (and Multimedia Appendices 1-7).
