# Supplementary material for: Digital Health Interventions for Chronic Wound Management: A Systematic Review and Meta-Analysis
Source: J Med Internet Res. 2024 Jul 16;26:e47904. doi: 10.2196/47904 (PMC11289581; doi:10.2196/47904)
Supplement: Multimedia Appendix 2 [file jmir_v26i1e47904_app2.docx]

Multimedia Appendix 2.

Search strategy for all databases.

**1. PubMed**

| Search | Query |
| --- | --- |
| #1 | “Digital technology” [Mesh] |
| #2 | “Telemedicine” [Mesh] |
| #3 | Mobile health* [Title/Abstract] OR Telehealth* [Title/Abstract] OR mHealth* [Title/Abstract] OR Telemedicine [Title/Abstract] OR Mobile phone [Title/Abstract] OR Mobile applications* [Title/Abstract] OR eHealth [Title/Abstract] OR Wearable technolog* [Title/Abstract] OR digital platform [Title/Abstract] OR online platform [Title/Abstract] OR Mobile platform [Title/Abstract] |
| #4 | “Pressure Ulcer” [Mesh] |
| #5 | Wound [Title/Abstract] OR Chronic wound [Title/Abstract] OR Pressure Ulcer*[Title/Abstract] OR Bedsore*[Title/Abstract] OR Pressure Sore*[Title/Abstract] OR Bed sore*[Title/Abstract] OR Decubitus Ulcer*[Title/Abstract] OR Pressure injur*[Title/Abstract] OR Ulcer* pressure[Title/Abstract] OR sore* bed[Title/Abstract] OR Venous ulcer*[Title/Abstract] OR Varicose Ulcer*[Title/Abstract] OR venous leg ulcer*[Title/Abstract] OR Arterial ulcer*[Title/Abstract] OR Diabetic Foot[Title/Abstract] OR Foot disease[Title/Abstract] OR Foot ulcer*[Title/Abstract] |
| #6 | #1 OR #2 OR #3 |
| #7 | #4 OR #5 |
| #8 | #6 AND #7 |

**2.Web of Science**

| Search | Query |
| --- | --- |
| #1 | TS= (digital health) |
| #2 | TI= (Mobile health* OR Telehealth* OR mHealth* OR Telemedicine OR Mobile phone OR Mobile Applications* OR eHealth OR Wearable technolog* OR digital platform OR Mobile platform OR online platform) |
| #3 | TS= (pressure ulcer) |
| #4 | ((((((((((((((((TI=(Wound )) OR TI=(chronic wound)) OR TI=(Pressure Ulcer*)) OR TI=(Bedsore*)) OR TI=(Pressure Sore*)) OR TI=(Bed sore*)) OR TI=(Decubitus Ulcer*)) OR TI=(Pressure injur*)) OR TI=(Ulcer* pressure)) OR TI=(sore* bed)) OR TI=(Venous ulcer*)) OR TI=(Varicose Ulcer*)) OR TI=(venous leg ulcer*)) OR TI=(Arterial ulcer*)) OR TI=(Diabetic Foot)) OR TI=(Foot disease)) OR TI=(Foot ulcer*) |
| #5 | #1 OR #2 |
| #6 | #3 OR #4 |
| #7 | #5 AND #6 |

**3 EMBASE**

| Search | Query |
| --- | --- |
| #1 | 'digital health':ab,ti OR 'mobile health*':ab,ti OR 'mHealth*':ab,ti OR 'telehealth*':ab,ti OR 'telemedicine':ab,ti OR 'mobile phone':ab,ti OR 'mobile applications*':ab,ti OR 'eHealth':ab,ti OR 'wearable technolog*':ab,ti OR 'digital platform':ab,ti OR 'online platform':ab,ti OR 'mobile platform':ab,ti |
| #2 | 'wound':ab,ti OR 'chronic wound':ab,ti OR 'pressure ulcer*':ab,ti OR 'bedsore*':ab,ti OR 'bed sore*':ab,ti OR 'pressure sore*':ab,ti OR 'decubitus ulcer*':ab,ti OR 'pressure injur*':ab,ti OR 'venous ulcer*':ab,ti OR 'varicose ulcer*':ab,ti OR 'venous leg ulcer*':ab,ti OR 'arterial ulcer*':ab,ti OR 'diabetic foot':ab,ti OR 'foot disease':ab,ti OR 'Foot ulcer*':ab,ti |
| #3 | #1 AND #2 |

**4 Cochrane Library**

| Search | Query |
| --- | --- |
| #1 | (digital health):ti,ab,kw OR (digital technology):ti,ab,kw OR (digital health intervention):ti,ab,kw |
| #2 | (Mobile health*):ti,ab,kw OR (Telehealth*):ti,ab,kw OR (mHealth*):ti,ab,kw OR (Telemedcine):ti,ab,kw OR (Mobile phone):ti,ab,kw OR (Mobile Applications*):ti,ab,kw OR (Wearable technolog*):ti,ab,kw OR (digital platform):ti,ab,kw OR (mobile platform):ti,ab,kw OR (online platfom):ti,ab,kw |
| #3 | (Wound OR Chronic wound OR Pressure Ulcer* OR Bedsore* OR Pressure Sore* OR Bed Sore* OR Decubitus Ulcer* OR Pressure injur* OR ulcer* pressure OR sore* bed OR Venous Ulcer* OR Varicose Ulcer* OR venous leg ulcer* OR Arterial ulcer* OR Diabetic Foot OR Foot Diseases OR Foot Ulcer*):ti,ab,kw |
| #4 | #1 OR #2 |
| #5 | #3 AND #4 |

**5 CINAHL**

| Search | Query |
| --- | --- |
| S1 | MJ digital health |
| S2 | MJ mobile health |
| S3 | MJ telemedicine or telehealth |
| S4 | TI digital health OR TI mobile health* OR TI telehealth* OR TI mHealth* OR TI Mobile Applications* OR TI Mobile phone OR TI Wearable technolog* OR TI digital platform OR TI online platform OR TI mobile platform |
| S5 | MJ pressure ulcer |
| S6 | TI wound OR TI chronic wound OR TI Pressure Ulcer* OR TI ( Bedsore* or Bed Sore* ) OR TI ( pressure sore or pressure ulcer or pressure damage or bed sores ) OR TI ( diabetic foot or diabetic feet or foot ulcer ) OR TI decubitus ulcer* OR TI ( venous ulcer or venous ulcers ) OR TI varicose ulcer* |
| S7 | S1 OR S2 OR S3 OR S4 |
| S8 | S5 OR S6 |
| S9 | S7 AND S8 |

**6 CNKI**

| Search | Query |
| --- | --- |
| #1 | （篇关摘）慢性伤口 + 压力性损伤 + 压力性溃疡 + 压疮 + 褥疮 + 糖尿病足溃疡 + 下肢溃疡 + 下肢静脉溃疡 + 动脉溃疡 |
| #2 | （篇关摘）数字健康 + 数字技术 + 移动健康 + 在线平台 + 可穿戴设备 + 移动手机 + 邮件 + 应用程序 + 远程医疗 + 远程监测 |
| #3 | #1 AND #2 |

**7 Wan Fang database**

| Search | Query |
| --- | --- |
| #1 | 主题:(数字健康) or 题名或关键词:(数字健康 OR 数字技术 OR 移动健康 OR 在线平台 OR 可穿戴设备 OR 移动手机 OR 邮件 OR 应用程序 OR 远程医疗 OR 远程监测) |
| #2 | 题名或关键词:(慢性伤口 OR 压力性损伤 OR 压力性溃疡 OR 压疮 OR 褥疮 OR 糖尿病足溃疡 OR 下肢溃疡 OR 下肢静脉溃疡 OR 动脉溃疡) |
| #3 | #1 AND #2 |

**8 VIP database**

| Search | Query |
| --- | --- |
| #1 | M=(数字健康 OR 数字技术 OR移动健康 OR 远程医疗 OR 远程监测 OR 移动手机 OR可穿戴设备OR 应用程序 OR 数字平台 OR 在线平台 OR 移动平台) |
| #2 | M=(慢性伤口 OR 压力性损伤 OR 压力性溃疡 OR 压疮 OR 褥疮 OR 糖尿病足 OR 足溃疡 OR 下肢溃疡 OR 静脉溃疡 OR 动脉溃疡) |
| #3 | #1 AND #2 |

**9 SinoMed**

| Search | Query |
| --- | --- |
| #1 | ( "数字健康"[常用字段:智能] OR "数字技术"[常用字段:智能] OR "移动健康"[常用字段:智能] OR "移动医疗"[常用字段:智能] OR "远程医疗"[常用字段:智能] OR "移动电话"[常用字段:智能] OR "可穿戴"[常用字段:智能] OR "移动应用"[常用字段:智能] OR "mHealth"[常用字段:智能] OR "数字平台"[常用字段:智能] OR "移动平台"[常用字段:智能] OR "在线平台"[常用字段:智能]) |
| #2 | ( "压力性损伤"[常用字段:智能] OR "压力性溃疡"[常用字段:智能] OR "压疮"[常用字段:智能] OR "褥疮"[常用字段:智能] OR "糖尿病足"[常用字段:智能] OR "足溃疡"[常用字段:智能] OR "静脉溃疡"[常用字段:智能] OR "动脉溃疡"[常用字段:智能]) OR "慢性伤口"[常用字段:智能] |
| #3 | #1 AND #2 |
