# Supplementary material for: Digital Health Interventions for Chronic Wound Management: A Systematic Review and Meta-Analysis
Source: J Med Internet Res. 2024 Jul 16;26:e47904. doi: 10.2196/47904 (PMC11289581; doi:10.2196/47904)
Supplement: Multimedia Appendix 3 [file jmir_v26i1e47904_app3.docx]

Multimedia Appendix 3.

Table S1. Characteristics of the included studies.

| Author | Year | Country | Wound etiology | No. of patients（T/C） | Intervention type; Provider | | Comparator | Follow-up (months) | Outcomes |
| --- | --- | --- | --- | --- | --- | --- | --- | --- | --- |
| Santamaria et al [47] | 2004 | Australia | Any etiology | 50/43 | Telemedicine (remote consultation); Wound care consultants + Nurses | | Usual care (local clinic wound care clinician) | 12 | ⑤⑥ |
| Wilbright et al [59] | 2004 | USA | Diabetic foot ulcer | 20/120 | Telemedicine (real-time interaction video); Nurses | | Usual care (Medical center on-site) | 3 | ①② |
| Terry et al [48] | 2009 | USA | Any etiology ^a^ | 62/98 | Telemedicine (remote consultation); Wound care specialists (WCSs) | | Usual care | 16 | ①⑦ |
| Vowden et al [22] | 2013 | UK | Any etiology | 23/11 | Telemedicine (remote consultation); Nursing home staff | | Usual care (home care) | 6 | ①⑥ |
| Stern et al [23] | 2014 | Canada | Pressure injuries | 93/131 | Follow-up by telephone and email; Multidisciplinary wound care team | | Usual care | 12 | ①⑥⑦ |
| Rasmussen et al [49] | 2015 | Denmark | Diabetic foot ulcer | 193/181 | Telemedicine (remote consultation); Nurses + Physicians | | Usual care (outpatient consultation) | 12 | ①⑤⑥⑦ |
| Zarchi et al [21] | 2015 | Denmark | Any etiology ^b^ | 50/40 | Telemedicine (web-based program); Wound care specialists | | Usual care (home care) | 12 | ①⑥ |
| Table S1. *Continued* | | | | | | | | | |
| Author | Year | Country | Wound etiology | No. of patients（T/C） | Intervention type; Provider | | Comparator | Follow-up (months) | Outcomes |
| Gao et al [24] | 2016 | China | Pressure injuries | 266/449 | Digital platform; Nurses | | Usual care | — | ⑨ |
| Zhang et al [50] | 2016 | China | Lower extremity venous ulcers | 20/20 | Telemedicine (remote consultation); Nurses | | Usual care | — | ① |
| Bergersen et al [60] | 2016 | Norway | Any etiology | 32/21 | Telemedicine (wound support network); Nurses | | Usual care (primary care) | 3 | ①③⑦ |
| Arora et al [51] | 2017 | Australia | Pressure injuries | 57/58 | Follow-up by telephone and email; Healthcare professional | | Usual care | 3 | ③④⑦ |
| Hu et al [64] | 2018 | China | Pressure injuries | 339/292 | Digital platform; Nurses | | Usual care | — | ⑨ |
| Le Goff-Pronost et al [61] | 2018 | France | Any etiology | 77/39 | Telemedicine (video conference); General practitioner + Nurses | | Usual care (primary care) | 9 | ①②⑥⑦ |
| Smith-Strøm et al [52] | 2018 | Norway | Diabetic foot ulcer | 94/88 | Follow-up by telephone; Nurses | | Usual care (outpatient consultation) | 12 | ①②⑤⑥⑧ |
| Wickstrom et al [62] | 2018 | Sweden | Any etiology | 100/1888 | Telemedicine (video consultation); Nurses | | Usual care (primary care) | 24 | ①②⑤⑥ |
| Wu et al [65] | 2019 | China | Pressure injuries | 588/588 | Digital platform; Nurses | | Usual care | — | ①⑤⑨ |
| Table S1. *Continued* | | | | | | | | | |
| Author | Year | Country | Wound etiology | No. of patients（T/C） | Intervention type; Provider | | Comparator | Follow-up (months) | Outcomes |
| Xie et al [66] | 2019 | China | Any etiology | 78/72 | Digital platform; Nurses | | Usual care | — | ④⑧ |
| Gamus et al [63] | 2019 | Israel | Any etiology | 277/373 | Telemedicine (video conference); Nurses | | Usual care (outpatient consultation) | 35 | ① |
| Fen et al [25] | 2020 | China | Pressure injuries | 74/169 | Digital platform; Nurses | | Usual care | — | ① |
| Teot et al [53] | 2020 | France | Any etiology ^c^ | 89/94 | Telemedicine (remote consultation); Wound care experts | | Usual care (home care + outpatient clinic) | 12 | ①②⑥⑦ |
| Zhou et al [54] | 2021 | China | Pressure injuries | 60/60 | Telemedicine (remote consultation); Nurses | | Usual care (hospital-community) | — | ④ |
| Shen et al [55] | 2022 | China | Any etiology | 48/48 | Digital platform; Nurses | | Usual care | — | ② |
| Wu et al [56] | 2022 | China | Any etiology | 158/158 | Digital platform; Nurses | | Usual care | — | ⑧ |
| Irgens et al [57] | 2022 | Norway | Pressure injuries | 28/28 | Telemedicine (remote consultation); Multidisciplinary wound team | | Usual care | 12 | ①②③ |
| Table S1. *Continued* | | | | | | | | | |
| Author | Year | Country | Wound etiology | No. of patients（T/C） | Intervention type; Provider | Comparator | | Follow-up (months) | Outcomes |
| Dardari et al [58] | 2023 | France | Diabetic foot ulcer | 90/90 | Telemedicine (via telemedicine software); Nurses | | Usual care | 12 | ①②⑤⑥⑦ |

^a^ Non-healing surgical wound, stasis ulcer, or pressure injuries.

^b^ Surgical wounds, pressure injuries, and cancer wounds excluded.

^c^ Pressure injuries, diabetic foot ulcers, leg ulcers, trauma, and postoperative wounds.

Outcomes:①wound healing;②wound healing time;③wound size;④PUSH-score;⑤adverse events;⑥all-cause mortality;⑦cost analysis;⑧patient satisfaction;⑨wound reporting rate.
