# Supplementary material for: Digital Health Interventions for Chronic Wound Management: A Systematic Review and Meta-Analysis
Source: J Med Internet Res. 2024 Jul 16;26:e47904. doi: 10.2196/47904 (PMC11289581; doi:10.2196/47904)
Supplement: Multimedia Appendix 4 [file jmir_v26i1e47904_app4.docx]

Multimedia Appendix 4.

Table S2. Risk bias assessment of the included RCTs.

| **Study** | **Selection bias** | |  | **Performance bias** |  | **Detection bias** |  | **Attrition bias** |  | **Reporting bias** |  | **Other bias** |
| --- | --- | --- | --- | --- | --- | --- | --- | --- | --- | --- | --- | --- |
|  | **Item1** | **Item2** |  | **Item3** |  | **Item4** |  | **Item5** |  | **Item6** |  |  |
| Arora et al. 2017 | Low | Low |  | Low |  | Low |  | Low |  | Low |  | Unclear |
| Irgens et al. 2022 | Low | Low |  | Low |  | Low |  | Low |  | Low |  | Unclear |
| Rasmussen et al. 2015 | Low | Low |  | Low |  | Low |  | Low |  | Low |  | Low |
| Santamaria et al. 2004 | Unclear | Unclear |  | Low |  | Low |  | Unclear |  | Unclear |  | Unclear |
| Shen et al. 2022 | Low | Low |  | Low |  | Low |  | Low |  | Low |  | Unclear |
| Smith-Strøm et al. 2018 | Low | Low |  | Low |  | Low |  | Low |  | Unclear |  | High |
| Stern et al. 2014 | Low | Low |  | Low |  | Low |  | Low |  | Unclear |  | Unclear |
| Teot et al. 2020 | High | High |  | Low |  | Low |  | Low |  | Unclear |  | High |
| Terry et al. 2009 | Unclear | Low |  | Low |  | Low |  | Unclear |  | Unclear |  | High |
| Vowden et al. 2013 | Low | Low |  | Low |  | Low |  | Unclear |  | Unclear |  | High |
| Wu et al. 2022 | Unclear | Unclear |  | Low |  | Low |  | Low |  | Low |  | Unclear |
| Zhang et al. 2016 | Low | Low |  | Low |  | Low |  | Low |  | Low |  | Unclear |
| Zhou et al. 2021 | Unclear | Unclear |  | Low |  | Low |  | Low |  | Low |  | Unclear |
| Dardari et al. 2023 | Low | Low |  | Low |  | Low |  | Low |  | Unclear |  | Low |
| Notes: Items: Item1, Random sequence generation. Item2, Allocation concealment. Item3, Blinding of participants and personnel. Item4, Blinding of outcome assessment. Item5, Incomplete outcome data. Item6, Selective reporting. | | | | | | | | | | | | |

Table S3. Risk bias assessment of the included quasi-experimental studies.

| **Study** | **Q1** | **Q2** | **Q3** | **Q4** | **Q5** | **Q6** | **Q7** | **Q8** | **Q9** |
| --- | --- | --- | --- | --- | --- | --- | --- | --- | --- |
| Gao et al. 2016 | Yes | Not applicable | Unclear | No | Yes | Not applicable | Yes | Yes | Yes |
| Hu et al. 2018 | Yes | Yes | Unclear | Yes | Yes | Not applicable | Yes | Yes | Yes |
| Wu et al. 2019 | Yes | Not applicable | Unclear | No | Yes | Not applicable | Yes | Yes | Yes |
| Xie et al. 2019 | Yes | Unclear | Unclear | Yes | Yes | Not applicable | Yes | Yes | Yes |
| Feng et al. 2020 | Yes | Yes | Unclear | Yes | Yes | Not applicable | Yes | Yes | Yes |
| Notes: Questions: Q1, Is it clear in the study what is the cause and what is the effect? Q2, Were the participants included in any similar comparisons? Q3, Were the participants included in any comparisons receiving similar treatment/care other than the exposure or intervention of interest? Q4, Was there a control group? Q5, Were there multiple measurements of the outcome both before and after the intervention/exposure? Q6, Was follow-up complete and, if not, were differences between groups in terms of their follow-up adequately described and analyzed? Q7, Were the outcomes of participants included in any comparisons measured in the same way? Q8, Were outcomes measured in a reliable way? Q9, Was appropriate statistical analysis used? | | | | | | | | | |

Table S4. Risk bias assessment of the included cohort studies.

| **Study** | **Bias due to confounding** | **Selection bias** | **Bias in classification of interventions** | | **Bias due to deviations from intended interventions** | **Bias due to missing data** | **Bias in measurement of outcomes** | **Bias in selection of reported result** | **Overall bias** |
| --- | --- | --- | --- | --- | --- | --- | --- | --- | --- |
| Wilbright et al. 2004 | Serious | Low | Low | Low | | Moderate | Low | Moderate | Serious |
| Zarchi et al. 2015 | Moderate | Low | Low | Low | | Moderate | Low | Low | Moderate |
| Bergersen et al. 2016 | Moderate | Low | Low | Low | | Moderate | Low | Moderate | Moderate |
| Le Goff-Pronost et al. 2018 | Serious | Low | Low | Low | | Moderate | Low | Moderate | Serious |
| Wickstrom et al. 2018 | Serious | Low | Low | Low | | Moderate | Low | Moderate | Serious |
| Gamus et al. 2019 | Moderate | Serious | Low | Moderate | | Serious | Low | Low | Serious |
