# Supplementary material for: Digital Health Interventions for Chronic Wound Management: A Systematic Review and Meta-Analysis
Source: J Med Internet Res. 2024 Jul 16;26:e47904. doi: 10.2196/47904 (PMC11289581; doi:10.2196/47904)

Study or Subgroup	Experimental Events	Experimental Total	Control Events	Control Total	Weight	Risk Ratio M-H, Random, 95% CI
**1.4.1 Randomized controlled trial**						
Zhang 2016	2	20	0	20	9.8%	5.00 [0.26, 98.00]
**Subtotal (95% CI)**		**20**		**20**	**9.8%**	**5.00 [0.26, 98.00]**
Total events	2		0			
Heterogeneity: Not applicable						
Test for overall effect: Z = 1.06 (P = 0.29)						
**1.4.2 Cohort studies**						
Bergersen 2016	15	32	5	21	38.7%	1.97 [0.84, 4.60]
Wilbright 2004	17	20	114	120	51.4%	0.89 [0.74, 1.08]
**Subtotal (95% CI)**		**52**		**141**	**90.2%**	**1.24 [0.47, 3.30]**
Total events	32		119			
Heterogeneity: $Tau^2 = 0.42$; $Chi^2 = 5.22$, df = 1 (P = 0.02); $I^2 = 81\%$						
Test for overall effect: Z = 0.43 (P = 0.67)						
**Total (95% CI)**		**72**		**161**	**100.0%**	**1.44 [0.51, 4.05]**
Total events	34		119			
Heterogeneity: $Tau^2 = 0.53$; $Chi^2 = 7.86$, df = 2 (P = 0.02); $I^2 = 75\%$						
Test for overall effect: Z = 0.69 (P = 0.49)						
Test for subgroup differences: $Chi^2 = 0.76$, df = 1 (P = 0.38), $I^2 = 0\%$						

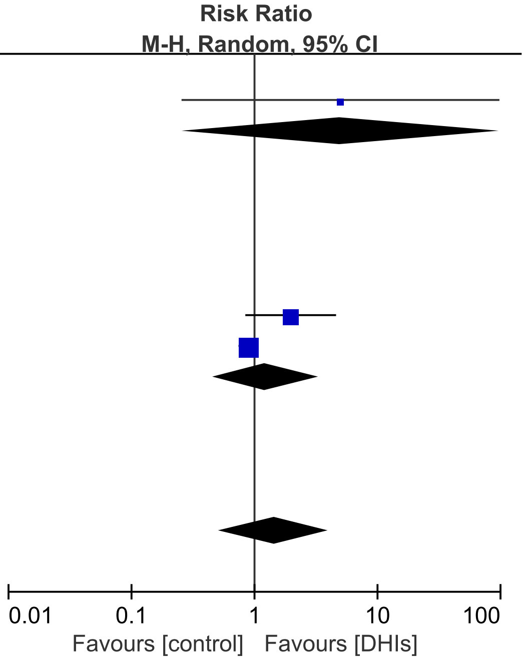

Supplement: Multimedia Appendix 5 [file jmir_v26i1e47904_app5.pdf]
