# Supplementary figures and images for: Digital Health Interventions for Chronic Wound Management: A Systematic Review and Meta-Analysis
Source: J Med Internet Res. 2024 Jul 16;26:e47904. doi: 10.2196/47904 (PMC11289581; doi:10.2196/47904)

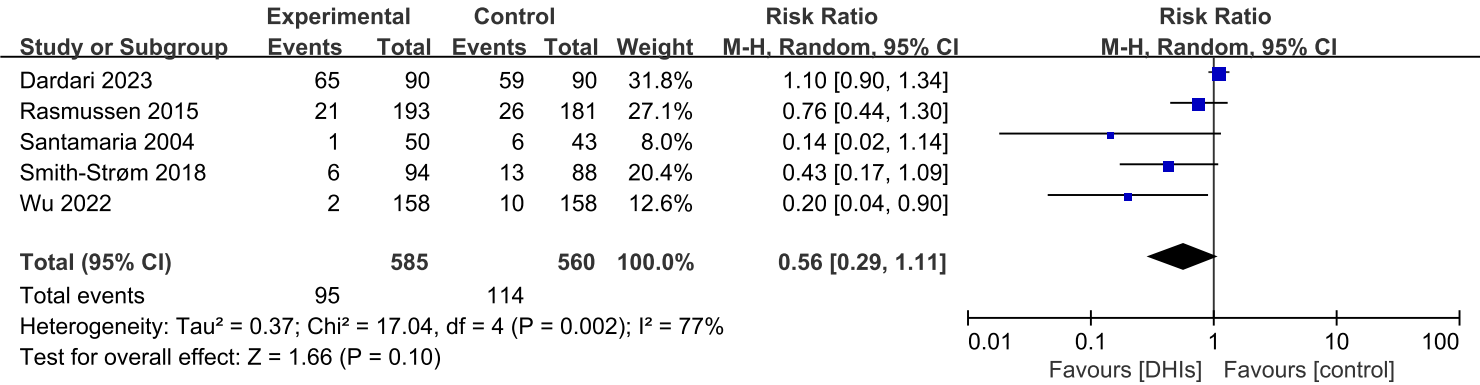

Supplement: Multimedia Appendix 6 [file jmir_v26i1e47904_app6.pdf]

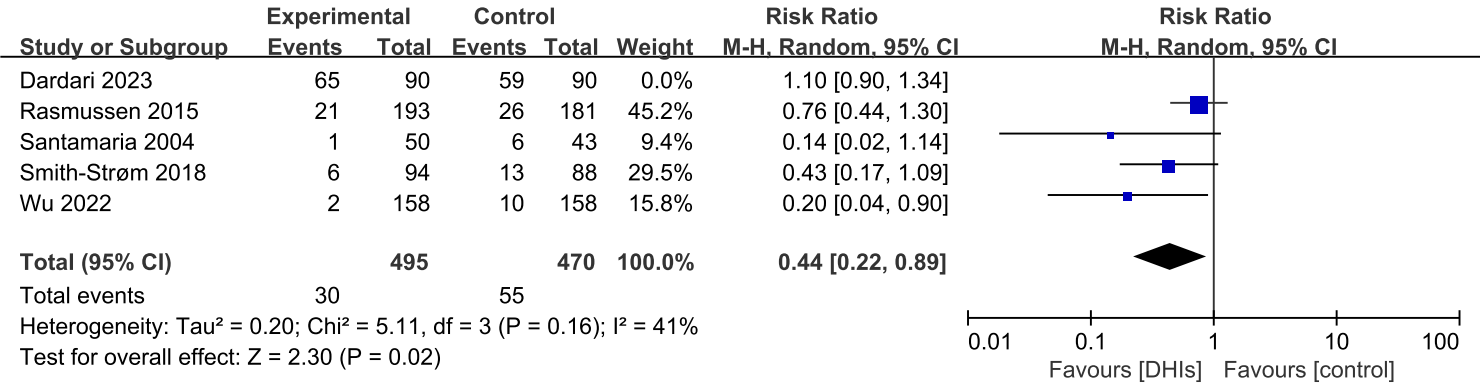

Supplement: Multimedia Appendix 7 [file jmir_v26i1e47904_app7.pdf]
